# Histopathologic Oral Cancer Prediction Using Oral Squamous Cell Carcinoma Biopsy Empowered with Transfer Learning

**DOI:** 10.3390/s22103833

**Published:** 2022-05-18

**Authors:** Atta-ur Rahman, Abdullah Alqahtani, Nahier Aldhafferi, Muhammad Umar Nasir, Muhammad Farhan Khan, Muhammad Adnan Khan, Amir Mosavi

**Affiliations:** 1 Department of Computer Science (CS), College of Computer Science and Information Technology (CCSIT), Imam Abdulrahman Bin Faisal University (IAU), P.O. Box 1982, Dammam 31441, Saudi Arabia; aaurrahman@iau.edu.sa; 2 Department of Computer Information Systems (CIS), College of Computer Science and Information Technology (CCSIT), Imam Abdulrahman Bin Faisal University (IAU), P.O. Box 1982, Dammam 31441, Saudi Arabia; aamqahtani@iau.edu.sa (A.A.); naldhafeeri@iau.edu.sa (N.A.); 3 Faculty of Computing, Riphah School of Computing and Innovation, Riphah International University, Lahore Campus, Lahore 54000, Pakistan; m.nasir@riphah.edu.pk; 4 Department of Forensic Sciences, University of Health Sciences, Lahore 54000, Pakistan; mfarhankhan1987@uhs.edu.pk; 5 Department of Software, Gachon University, Seongnam 13120, Korea; 6 John von Neumann Faculty of Informatics, Obuda University, 1034 Budapest, Hungary; mosavi.amirhosein@uni-nke.hu; 7 Institute of Information Engineering, Automation and Mathematics, Slovak University of Technology in Bratislava, 81107 Bratislava, Slovakia; 8 Faculty of Civil Engineering, TU-Dresden, 01062 Dresden, Germany

**Keywords:** oral cancer, oral squamous cell carcinoma, transfer learning, neural network, AlexNet, medical imaging, machine learning, angiogenic, malignant, artificial intelligence

## Abstract

Oral cancer is a dangerous and extensive cancer with a high death ratio. Oral cancer is the most usual cancer in the world, with more than 300,335 deaths every year. The cancerous tumor appears in the neck, oral glands, face, and mouth. To overcome this dangerous cancer, there are many ways to detect like a biopsy, in which small chunks of tissues are taken from the mouth and tested under a secure and hygienic microscope. However, microscope results of tissues to detect oral cancer are not up to the mark, a microscope cannot easily identify the cancerous cells and normal cells. Detection of cancerous cells using microscopic biopsy images helps in allaying and predicting the issues and gives better results if biologically approaches apply accurately for the prediction of cancerous cells, but during the physical examinations microscopic biopsy images for cancer detection there are major chances for human error and mistake. So, with the development of technology deep learning algorithms plays a major role in medical image diagnosing. Deep learning algorithms are efficiently developed to predict breast cancer, oral cancer, lung cancer, or any other type of medical image. In this study, the proposed model of transfer learning model using AlexNet in the convolutional neural network to extract rank features from oral squamous cell carcinoma (OSCC) biopsy images to train the model. Simulation results have shown that the proposed model achieved higher classification accuracy 97.66% and 90.06% of training and testing, respectively.

## 1. Introduction

Oral cancer is one of the most frequent deadly diseases and has long been a serious public health concern across the world. Oral cancer is a subset of head and neck malignancies, with 475,000 new cases diagnosed each year globally. Early-stage sickness has a survival rate of about 80%, whereas late-stage sickness has a survival rate of less than 20% [1,2]. Squamous cell carcinoma of the oral cavity is the most prevalent form of oral cancer, accounting for more than 85% of cases. Although early detection of oral cancer is critical, most patients are identified at the last stage of the illness, resulting in a dismal prognosis. 

Due to the clinical appearance of oral cancer being insufficient to identify the dysplastic state, analysis, or degree, therapy selection based on the clinical appearance of the illness is insufficient.

Oral cancer is linked to a variety of risk factors, and the post-treatment survival rate is similarly unexpected [3,4]. Biological models, as well as medical forms of related and lesion-free tumor models, may be recognized in many regions of the body using appearance models and stereotypes without labeling. Potentially cancerous lesions like leukoplakia, erythroplasia, and oral submucosal fibrosis are also common in the at-risk group. It is also critical to distinguish between benign and malignant tumors. Age, gender, and smoking history can all have an impact on the prognosis of oral cancer [5]. Understanding the advancements of technology such as artificial intelligence may help to resolve any healthcare snags [6,7]. A sore or ulcer that does not heal and may cause discomfort or bleeding is the most prevalent indication of cancer. White or red sores in the mouth, lips, gums, or tongue that are not healing, a lump or mass in the mouth, loose teeth, chewing or swallowing difficulties, jaw swelling, trouble talking, and persistent painful throat [8]. The use of Artificial Intelligence in the treatment of oral malignant growths has the potential to address existing problems in disease detection and prognosis prediction. Artificial intelligence, which replicates human cognitive capabilities, is a technological achievement that has captured the imaginations of scientists all around the world [9]. Its application in dentistry has only just begun, resulting in amazing results. The tale begins about 300 BC. C. Plato depicted an important model of brain activity. The artificial intelligence system is a framework that accepts information, discovers designs, trains using data, and generates outcomes [10,11]. Artificial intelligence operates in two stages: the first, which involves training, and the second, which includes testing. The parameters of the model set are determined by the training data. Data from prior instances, such as patient data or data from other examples, are used retrospectively by the model. The test phase is subsequently subjected to these criteria. Various biomarkers identified by artificial intelligence in various research have established prognostic variables for oral cancer. Early detection of a malignant tumor improves patient survival and treatment options [12]. Much research on medical image analysis for smartphone-based oral cancer detectors based on artificial intelligence algorithms has been done. Artificial intelligence technology makes it easier to diagnose, treat, and manage patients with oral cancer. To aid diagnosis, artificial intelligence decreases physician burden, complicated data, and exhaustion [13]. Given the practicality and alleged benefits of deep learning techniques in cancer prediction, their use in this field has gotten a lot of interest in recent years. This is because it is designed to assist doctors in making educated decisions, therefore enhancing and encouraging better patient health management. Surprisingly, technological advancements have resulted in the transition from neural networks to deep neural networks. This deep learning method has also been lauded for its potential to enhance cancer management.

## 2. Literature Review

The researchers’ objective was to diagnose the early stages of oral cancer utilizing the less accurate output of Naive Bayes, Multilayer Perceptron, KNN, and Support Vector Mechanical techniques. Oral cavities and analysis improve classification precision [14]. The researcher’s objective is to develop a model for doctors. Use tree-based decision-making approaches, artificial neural network vector maintenance methods, and DATA, NN, and HDM high precision analyses. The representation of the ADM’s score is the best suited for detecting breast cancer recurrence since it has the highest precision and the lowest error level. When both ANN and DT are evaluated, the results suggest that SVM is the best technique for diagnosis [15]. Madhura V, Meghana Nagaraju, and their colleague review several reports to investigate the diagnosis of oral cancer using machine learning [16]. They then utilize categorization rules to anticipate and association rules to demonstrate attribute co-dependency [17]. It then employs the a priori technique to pick frequent item sets and construct the association rule from the bottom up, employing a breadth search and hash to efficiently count the items. In their study, researchers utilize an adaptive fuzzy system based on deep neurons to get exact findings in data mining techniques. The procedure starts with data processing and grouping with Fuzzy C-Means. The architecture of an adaptive fuzzy system based on deep neurons has been presented, and analysis methods have been used to obtain correct findings such as precision, accuracy, and so on [18]. Their study made use of data sets including 251 X-rays from the equator, which were then subdivided into experimental data testing and training methods such as in-depth ANA investigations, transfer studies, and Convolutional Neural networks [19].

The researcher’s objective was to test novel automation ways for Oral Squamous Cell Carcinoma diagnoses on clear pictures utilizing in-depth training and Convolutional Neural Network methods. The focus of this Convolutional Neural Network is on the search for quotations, pictures, training, data, and evaluation [20]. Oral cancer can exhibit a wide range of patterns and behaviors [21]. In recent years, researchers used numerous machine learning techniques to overcome cancer [22], and the machine learning models can detect cancer. Machine learning predicts oral cancer is way better than previous prediction techniques [23]. Oral cancer is a fatal disease, and the major root of this fatal disease comes from the genome [24] and a variety of pathogenic changes [25]. Early prediction of oral cancer [26] and its treatment can increase the patient survival chances. Oral cancer is a progressive and very complicated disease [27] that can only predict using numerous machine and deep learning algorithms. So, the researchers provide the combining machine learning strategies [28] to predict oral cancer in its early stages. Researchers can use different histopathological machine learning [29] approaches to overcome cancer. Some researchers provide the fused machine learning-based solution [30] to predict cancer using real-time data and achieved good accuracy using different neural algorithms. Previous research used cloud-based deep learning approaches [31] to overcome cancerous diseases and gave better treatment to decrease the high mortality rate in females. Machine learning approaches help in genes association to overcome the cancerous empowered with deep learning approaches [32]. Researchers used digital images [33] to predict cancer using artificial neural networks and deep CNN [34] approaches to get highly efficient results. During the COVID phase, various researchers apply machine learning approaches to COVID patients to predict cancer [35] and get highly efficient results using different preprocessing techniques. Deep learning radionics-based detections [36] on cancerous patients give high feature results with the help of chemoradiotherapy.

The researcher’s objective was to test novel automation ways for Oral Squamous Cell Carcinoma diagnoses on clear pictures utilizing in-depth training and Convolutional Neural Network methods. The focus of this Convolutional Neural Network is on the search for citations, pictures, training, data, and classification. The researchers used machine learning techniques and genetic data to predict oral cancer development in OPL patients. To examine the course of oral cancer in individuals with a history of OPL, the researchers employed a Support Vector Machine, Multi-Layer Perceptron, a minimally invasive procedure, and a DNN [37]. The researcher utilized ordered electrical machines to classify the usage of hyperspectral to identify lung cancer in the amplification attempt. They divided the data into pictures using a Convolutional Neural Network. In this regard, in-depth research approaches were used to address the lack of an independent design of the cancer detection system [38].

The majority of the approaches need complex system configuration, resulting in significant operational expenses. The researchers assessed several cancer detection approaches and clarified the benefits of symptomatic simulations. As a result, HSI can be used to classify data. They use a vector support machine with a self-mapping structure to categorize the data [39].

Table 1 shows most of the previous research used machine and deep learning techniques to predict oral cancer using OSCC biopsies and other datasets, but they did not achieve the highest accuracy due to their highlighted weakness. As observed from previous studies oral cancer prediction is an important mission to save many lives. So, in this study transfer learning empowered model is proposed, which extract features and train on OSCC biopsy data to predict oral cancer.

## 3. Materials and Methods

**Figure 1:**
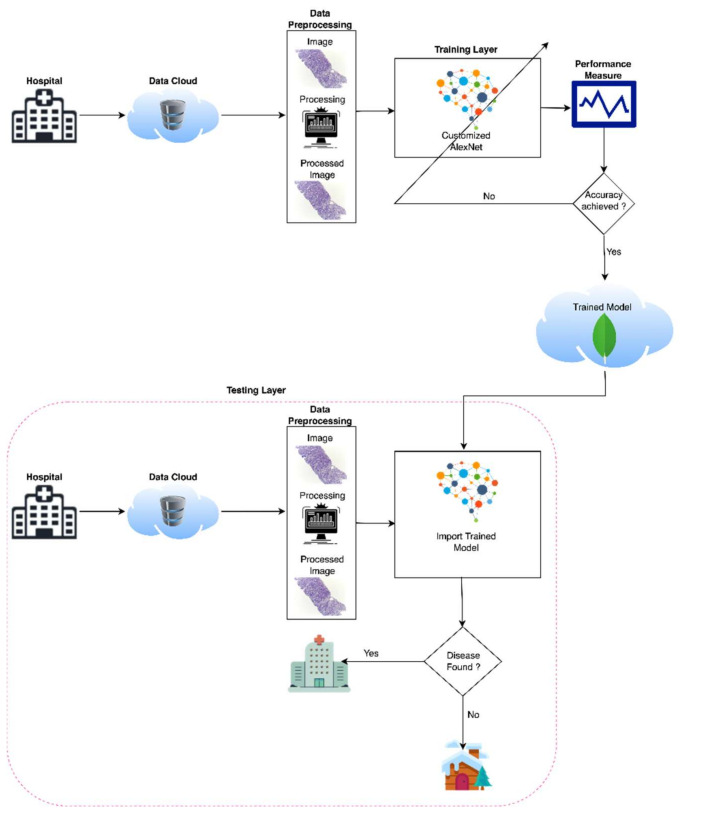
Proposed model of oral cancer prediction empowered with transfer learning.

With the development of machine learning and deep learning, every field of medical image diagnosing is very easy to analyze and predict with the help of medical images. There are various artificial intelligence techniques to diagnose medical diseases, but deep learning has efficient results with a very effective approach. So, in this study deep learning has a major role in the first stage prediction of medical images with a high prediction efficiency and authentic results. The proposed model used a MacBook Pro 2017 with 16 GB RAM used for simulation and used transfer learning to simulate the cancerous images. The proposed model of oral cancer prediction using Oral Squamous Cell Carcinoma biopsy empowered with transfer learning Figure 1 consists of three phases. In the first phase, the proposed model takes binary input (Oral Squamous Cell Carcinoma images and Normal Images) and transfers these inputs to the image pre-processing section. The image pre-processing section performs image dimension analysis to transform input images into 227 × 227 resolution of height and width, respectively, because the AlexNet model input layer requirement is 227 × 227-dimension images for the training model.

In the second phase, the proposed model gets a pre-trained model AlexNet of the neural network then the proposed model modifies AlexNet according to the research requirements using transfer and train model with pre-processed Oral Squamous Cell Carcinoma biopsy images and then stores this trained model into the cloud for easy access in testing at any time. In the third phase, which is known as the testing phase, the trained model retrieves from the cloud and apply to the neural network, and the neural network gets the input of pre-processed Oral Squamous Cell Carcinoma biopsy images to predict oral cancer. If the cancer is diagnosed then the patient will consult with paramedical staff otherwise, the patient has to be free to leave for home.

Table 2 shows the pseudocode of the proposed model for oral cancer prediction. After starting the pseudocode first, the proposed model gets input from the data cloud and preprocessed the data for customized AlexNet, and the code starts the training phase for training the model, and then in image testing phase pseudocode of the proposed model used trained model for the prediction of cancerous images. At the end, the code calculates the prediction accuracy and misclassification rate using various performance matrices.

## 4. Data Set

**Figure 2:**
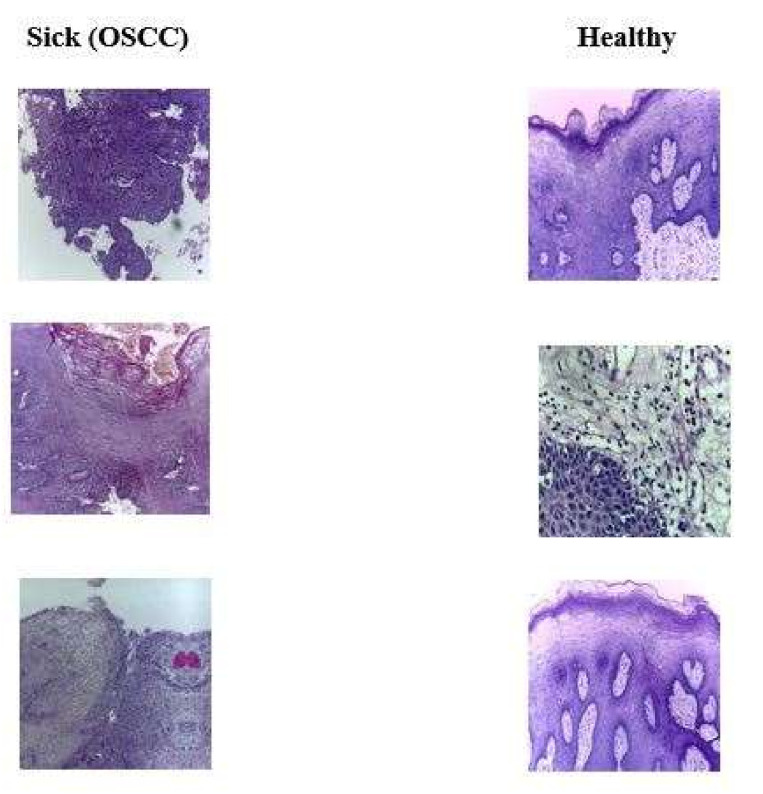
Sick and healthy oral squamous cell carcinoma biopsy dataset.

The Oral Squamous Cell Carcinoma (OSCC) biopsy data retrieve from Kaggle, which has open access to everyone [41]. This oral cancer dataset contains binary class one is sick with oral cancer, and the second class contains normal patients. The proposed model uses this dataset to analyze and predict oral cancer. Table 3 shows the detailed dataset classes, and Figure 2 shows the samples of both classes of the dataset.

## 5. Customized AlexNet

**Figure 3:**
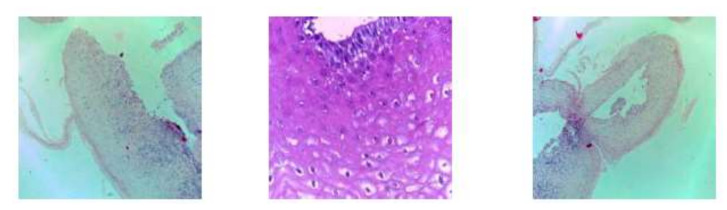
Pre-processed (227 × 227) oral squamous cell carcinoma biopsy images.

In this period, deep learning algorithms are very efficient to analyze medical images with the help of many pre-trained models for prediction. In this study, the proposed model used the pre-trained convolutional neural network model AlexNet for transfer learning to analyze and predict oral cancer using Oral Squamous Cell Carcinoma biopsy images. Customized AlexNet is known as the transfer learning technique and this technique is very primary and contiguous to analyzing and predicting medical image diagnosing. So, we customized the AlexNet algorithm according to input and prediction requirements. Customized AlexNet contains two fully connected layers, five max pool layers, and five convolutional layers. Each layer of AlexNet contains ReLU as an activation function. For the input layer of AlexNet, the firstly proposed model processed images into 227 × 227 resolution because the input layer of AlexNet only read this height and width, respectively. Figure 3 shows samples of pre-processed images in 227 × 227 resolution. 

**Figure 4:**
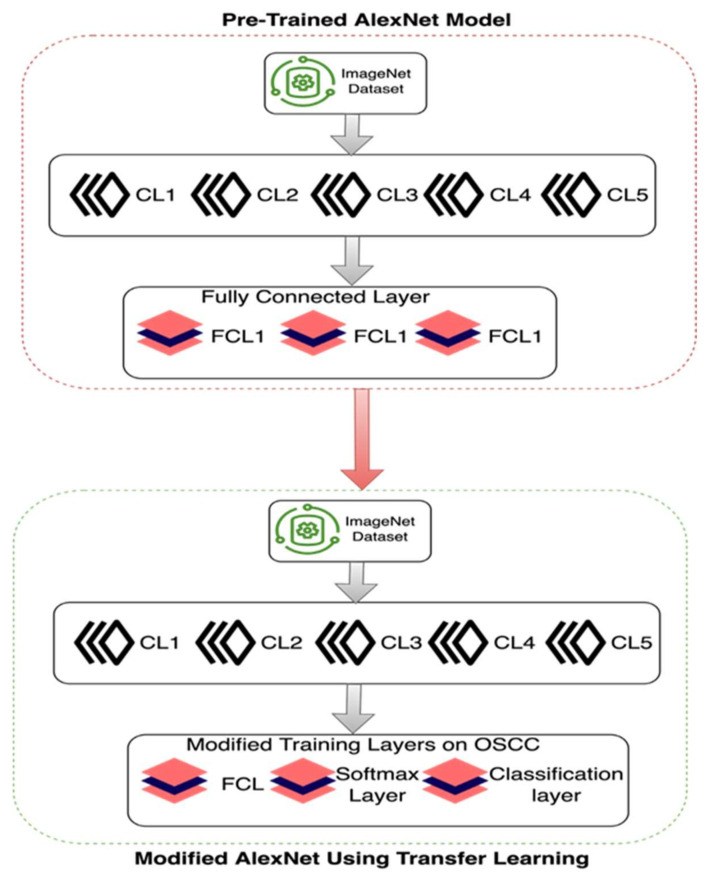
Customized AlexNet for oral cancer prediction using transfer learning.

Figure 4 shows the customized AlexNet model using transfer learning, in this customized AlexNet the last three layers of AlexNet were customized for analysis and prediction purposes. Input instances of all classes are fully connected. The output size of the fully connected layer is equal to the length of the total labels of the prediction class. The purpose of the Softmax layer is to detect the boundaries of input images that are represented in the convolutional layer, while the fully connected layer learns from all class features.

The purpose of the convolutional layer is to extract the features from input class images by using different image extract feature filters i-e gabber filter and secure the connection between image pixels. Each layer contains a ReLU activation function to activate neurons to weighted sum or not. So, the fully connected layer is set as binary for prediction purposes. So, the proposed model used this customized AlexNet model to train OSCC biopsy images to predict oral cancer.

## 6. Simulation and Results

In this study, the proposed model applied a customized pre-trained AlexNet model to predict oral cancer. MATLAB 2020a is used to simulate results on MAC Book Pro 2016 i5 with 16 GB RAM containing a high GPU. The proposed model split the dataset of oral cancer prediction into 70% for training and 30% for testing. After the testing phase, the proposed model used numerous performance statistical parameters [30,42,43,44,45,46,47] like Classification Accuracy (CA), Classification Miss Rate (CMR), Specificity, Sensitivity, F1-Score, Positive Predicted Value (PPV), Negative Predicted Value (NPV), False Positive Ratio (FPR), False Negative Ratio (FNR), Likelihood Positive Ratio (LPR), Likelihood Negative Ratio (LNR) and Fowlkes-Mallows Index to evaluate the results of oral cancer prediction. The proposed model represents Ç for predicted true positive values, ø for predicted true negative values, ∂ for predicted false positive values, and µ for predicted false negative values.

(1) $$\mathrm{CA}\;=\;\frac{Ç\;+\;ø\;}{Ç\;+\;ø\;+\partial +\;µ\;}\times 100$$

(2) $$\mathrm{CMR}\;=\;100-\left(\frac{Ç\;+\;ø\;}{Ç\;+\;ø\;+\partial +\;µ\;}\times 100\right)$$

(3) $$\mathrm{Sensitivity}\;=\;\frac{Ç}{Ç\;+µ\;}\times 100$$

(4) $$\mathrm{Specificity}\;=\;\frac{ø}{ø\;+\partial \;}\times 100$$

(5) $$F1\;\mathrm{score}\;=\;\frac{2\partial }{2\partial +\partial +\;µ\;}\times 100$$

(6) $$\mathrm{PPV}\;=\;\frac{Ç}{Ç\;+\partial \;}\times 100$$

(7) $$\mathrm{NPV}\;=\;\frac{ø}{ø\;+\;µ}\times 100$$

(8) $$\mathrm{FPR}\;=\;100-\left(\frac{ø}{ø\;+\partial \;}\times 100\right)$$

(9) $$\mathrm{FNR}\;=\;100-\left(\frac{Ç}{Ç\;+µ\;}\times 100\right)$$

(10) $$\mathrm{LPR}\;=\;\frac{\frac{Ç}{Ç\;+µ\;}\times 100}{100-\left(\frac{ø}{ø\;+\partial \;}\times 100\right)}$$

(11) $$\mathrm{LNR}\;=\;\frac{100-\left(\frac{Ç}{Ç\;+µ\;}\times 100\right)}{\frac{ø}{ø\;+\partial \;}*100}$$

(12) $$\mathrm{Fowlkes}-\mathrm{Mallows}\;\mathrm{Index}\;=\;\sqrt{\left(\frac{Ç}{Ç\;+\partial \;}\times 100\right)\times \left(\frac{Ç}{Ç\;+µ\;}\times 100\right)}$$

Table 4 shows the proposed model training simulation parameters results on the various number of tested epochs with the same layers settings 227 × 227 × 3, MAX of image dimension, and pooling method, respectively. The learning rate of every layer is set at 0.001. So, the proposed model shows that mini-batch loss at the final stage of the 70 epoch is 0.7336. Table 5 shows proposed model training simulation accuracies on every 10th epoch, and every epoch consists of 38 iterations. So, the proposed model achieved the highest training accuracy 97.66%, with 00:08:34 elapsed time on the 70th epoch.

**Figure 5:**
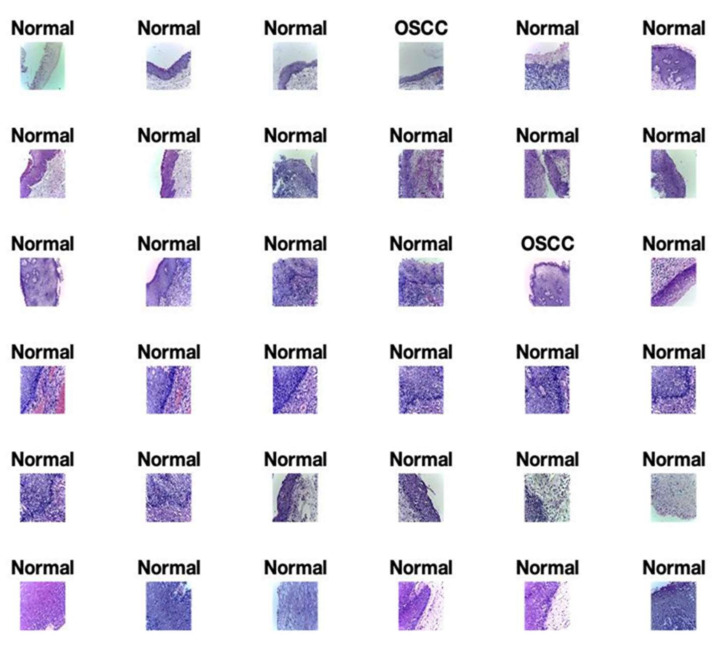
The proposed model predicted results of oral cancer during validation.

Figure 5 shows the final predicted results of Normal and oral cancer biopsies by the proposed model for oral cancer prediction. There are thousands of images that are not possible to portray in this study. So, the proposed model shows the first 36 results as a sample of tested results. These predicted images are generated using the proposed model for the prediction of oral cancer. Figure 5 shows the accurately predicted images of a cancerous cell labeled as “OSCC” and normal predicted cell images labeled as “Normal”.

**Figure 6:**
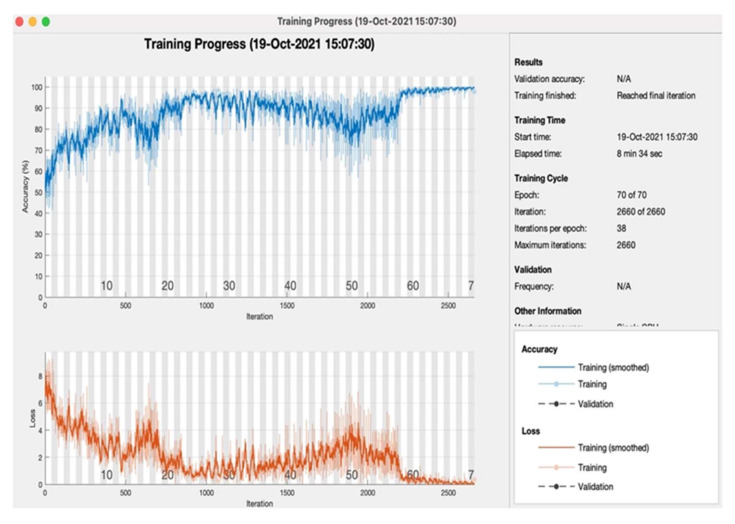
Proposed model of oral cancer prediction accuracy and loss with respect to iteration during training.

**Figure 7:**
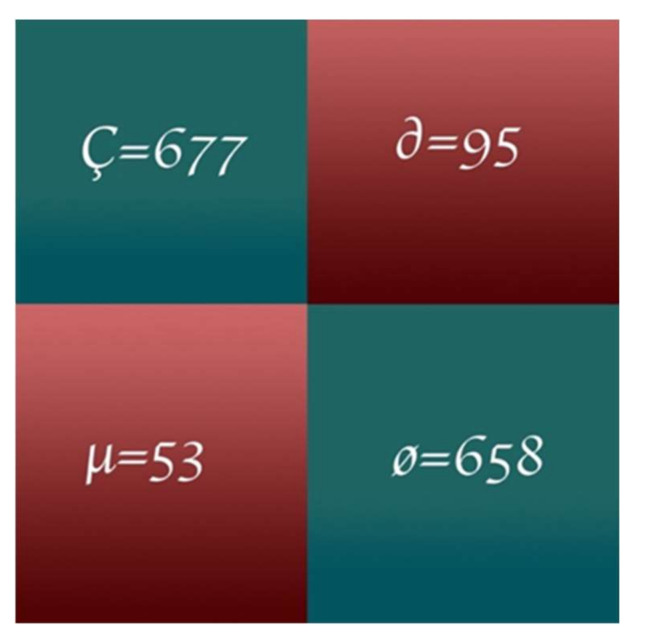
Proposed model testing confusion matrix.

Figure 6 shows the proposed model training progress on 70 epochs, as in the training progress shown proposed model converges after the 59th epoch, and at the 70th epoch proposed model achieved 97.66% accuracy with a 2.34% loss rate. Figure 7 shows the proposed model testing confusion matrix on oral cancer prediction. So, the confusion matrix consists of a total of 1483 instances for testing of class 1 “Normal” and class 2 “Oral Cancer”. As Figure 7 shows, the proposed model truly predicted 677 patients that are normal and 658 patients that have oral cancer. Furthermore, the proposed model wrong predicted 95 normal patients and 53 cancerous patients.

Table 6 shows the proposed performance parameter results empowered with transfer learning to analyze the performance of the model. Performance results of the proposed model are 90.02%, 9.08%, 92.74%, 87.38%, 90.15%, 87.69%, 92.55%, 12.62%, 7.26%, 7.35%, 0.08%, 90.18% of Classification Accuracy, Classification Miss Rate, Sensitivity, Specificity, F1-score, Positive Predicted Value, Negative Predicted Value, False Positive Ratio, False Negative Ratio, Likelihood Positive ratio, Likelihood Negative Ratio and Fowlkes-Mallows Index respectively. So, the proposed model of oral cancer prediction using Oral Squamous Cell Carcinoma biopsy empowered with transfer learning achieved the best prediction accuracy as compared with the previous studies because the proposed model covers the previous limitation e.g., used data image preprocessing layers and learning criteria accurately to achieve high prediction results with the at most reliability. With the help of data preprocessing, the proposed model achieved the best predicted results with the help of a customized AlexNet algorithm, customized layers of AlexNet play a pivotal role to get high prediction accuracy.

Table 7 shows the comparative analysis of the proposed model for oral cancer prediction using transfer learning with previous research. The proposed model performance in terms of classification accuracy during the testing phase. During the testing phase, the proposed model gives 90.06% and 9.94%, classification accuracy, and miss-Classification rate, respectively, and during the training phase the proposed model gives 97.6% and 2.34%, classification accuracy, and miss-rate respectively. The overall summary of the analysis shows that the classification accuracy is improved by around 12% as compared with the previously proposed models of machine learning [26,27] and deep learning [28]. The proposed model gives better classification accuracy as compared with the previously published research [26,27,28].

## 7. Conclusions

This research used customized AlexNet of CNN to predict cancerous oral and normal oral using Oral Squamous Cell Carcinoma biopsy images. The performance parameters like Classification Accuracy, Classification Miss Rate, Sensitivity, Specificity, F1-score, Positive Predicted Value, Negative Predicted Value, False Positive Ratio, False Negative Ratio, Likelihood Positive ratio, Likelihood Negative Ratio, and Fowlkes-Mallows Index were used to analyze the proposed model optimally and efficiently. With the help of statistical performance parameters, we can easily measure the reliability of the proposed model for the prediction of oral cancer. So, the proposed model achieved 90.06% and 9.08% of prediction accuracy and loss rate, respectively. The reason behind the highest accuracy achievement of the proposed model is its customized layer techniques, dataset preprocessing techniques, and training epochs. This study and the proposed model can be used in the medical field to prevent unnecessary treatments and tests, also paramedic staff can easily do the treatment of cancerous patients to prevent unnecessary deaths. The major contribution of this study in health 5.0 for early prediction and treatment of oral cancer. In the future, this proposal can be extended to fuse datasets and also can apply fuzzy techniques to get more precise results empowered with federated machine learning and blockchain for data security and fast prediction results.

The method is simple to use, affordable, and appropriate for oral cancer detection in impoverished nations. In a future study, scientists can utilize real-time data and a large database to enhance the transfer learning and machine learning structure. Using histopathological images, this research can also be used for the early diagnosis of different stages of oral squamous cancer detection. This will help doctors provide proper medicine and save lives.

## Figures and Tables

**Table 1:** Compare and weaknesses of previous studies.

Publication	Method	Dataset	Accuracy	Limitation
A.Alhazmi [26]	ANN	Public	78.95%	Requires data preprocessing
C.S. Chu [27]	SVM, KNN	Public	70.59%	Requires data preprocessing
R.A.Welikala [28]	ResNet101	Public	78.30%	Requires data preprocessing and learning criteria decision method
V. Shavlokhova [40]	CNN	Private	77.89%	Requires better image data preprocessing techniques and learning criteria method
M. Aberville [20]	Deep Learning	Public	80.01%	Requires data image preprocessing techniquesClass instances
H. Alkhadar [23]	KNN, Logistic Regression, Decision Tree, Random Forest	Public	76%	Requires handcrafted features

**Table 2:** Pseudocode of the proposed model for oral cancer prediction.

1	Start
2	Input Oral Cancer Data from Data Cloud
3	Pre-process Oral Cancer data
4	Load Data
5	Load Customized Model
6	Prediction of Oral Cancer using Transfer Learning (AlexNet)
7	Training Phase
8	Image Testing Phase
9	Compute the Performance and Accuracy of the proposed model by using the Performance Matrix
10	Finish

**Table 3:** OSCC biopsy dataset instances.

Classes	No. of Images
Sick (OSCC)	2511
Healthy	2435

**Table 4:** Proposed model training simulation parameters.

No. of Epochs	Learning Rate	No. of Layers	Image Dimension	Pooling Method	Mini-Batch Loss
**10**	0.001	25	227 × 227 × 3	MAX	2.5674
**20**					2.3498
**30**					1.3600
**40**					1.4948
**50**					6.1029
**60**					0.2491
**70**					0.3736

**Table 5:** Proposed model training simulation accuracies.

No. of Epochs	Learning Rate	Accuracy (%)	Loss Rate (%)	Iterations	Time Elapsed (hh:mm:ss)
10	0.001	76.12	23.88	38 per epoch	00:03:15
20		80.35	19.65		00:03:45
30		86.15	13.85		00:04:34
40		90.62	9.38		00:04:55
50		85.94	14.06		00:06:11
60		94.44	5.56		00:07:17
70		97.66	2.34		00:08:34

**Table 6:** Proposed model performance parameter results using transfer learning.

Instances (1483)	Testing (%)
**CA**	90.02
**CMR**	9.08
**Sensitivity**	92.74
**Specificity**	87.38
**F1-Score**	90.15
**PPV**	87.69
**NPV**	92.55
**FPR**	12.62
**FNR**	7.26
**LPR**	7.35
**LNR**	0.08
**FMI**	90.18

**Table 7:** Comparative analysis with previous research.

Work	Preprocessing Layer	Models	Classification Accuracy	Miss-Classification Rate
A.Alhazmi [26]	No	ANN	78.95%	21.05%
C.S. Chu [27]	No	SVM, KNN	70.59%	29.41%
R.A.Welikala [28]	No	ResNet101	78.30%	21.70%
**Proposed Model**	Yes	Transfer Learning (AlexNet)	90.06%	9.94%

## Data Availability

The simulation files/data used to support the findings of this study are available from the corresponding author upon request.
